# Relación entre la susceptibilidad a enfermedades neurodegenerativas y los factores genéticos en poblaciones expuestas al mercurio en Medellín, Colombia

**DOI:** 10.7705/biomedica.7634

**Published:** 2026-03-02

**Authors:** Efrén de Jesús Avendaño, Andrea Paola Castillo

**Affiliations:** 1 CBATA, Departamento de Ciencias Básicas y Áreas Comunes, Tecnológico de Antioquia Institución Universitaria, Medellín, Colombia Departamento de Ciencias Básicas y Áreas Comunes Tecnológico de Antioquia Institución Universitaria Medellín Colombia

**Keywords:** enfermedades neurodegenerativas, genes, alelos, haplotipos, mercurio, neurodegenerative diseases, genes, alleles, haplotypes, mercury

## Abstract

**Introducción.:**

Las enfermedades neurodegenerativas, por ejemplo la de Alzheimer y la de Parkinson, están asociadas con factores genéticos y ambientales como la exposición al mercurio, un metal pesado con efectos neurotóxicos.

**Objetivos.:**

Identificar y analizar los alelos y genes vinculados a las enfermedades neurodegenerativas, en relación con la exposición al mercurio en Colombia.

**Materiales y métodos.:**

Se recopiló información de la literatura científica y, también, genotipos poblacionales de bases de datos públicas, abarcando 94 adultos colombianos genotipificados bajo la referencia CLM *(Colombians from Medellín).* La trazabilidad de los datos se asegura mediante el registro ID en la base de datos del proyecto 1000 Genomas, garantizando el consentimiento informado y la aprobación bioética. Se analizaron 11 genes *(GSTP1, ATP7B, BDNF, GCLC, GCLM, MT1A, MT4, ABCC2, ABCB1, GPX1* y *GPX4)* con 18 polimorfismos distribuidos en 10 cromosomas, utilizando el programa SNPstats^TM^. Se aplicó la prueba de X^2^ para evaluar el equilibrio de Hardy-Weinberg, considerando significativas las desviaciones con p < 0,05.

**Resultados.:**

Los resultados demostraron una gran probabilidad de relación entre las enfermedades neurodegenerativas, como la de Alzheimer y la de Parkinson, y la exposición al mercurio en personas que tienen variantes génicas relacionadas con el metabolismo del glutatión o con las rutas metabólicas para el transporte y la excreción del mercurio.

**Conclusiones.:**

La bioacumulación de mercurio, el paso de la barrera hematoencefálica, la inflamación del sistema nervioso central y el estrés oxidativo por especies reactivas de oxígeno y nitrógeno, repercuten en las alteraciones genéticas o su expresión y aumentan la posibilidad de desarrollar las enfermedades de Alzheimer y Parkinson.

## Abstract (en)

**Introduction.:**

Neurodegenerative diseases such as Alzheimer's and Parkinson's are associated with genetic and environmental factors, including exposure to mercury, a heavy metal with neurotoxic effects.

**Objectives.:**

To identify and analyze alleles and genes linked to neurodegenerative diseases in relation to mercury exposure in Colombia.

**Materials and methods.:**

Scientific literature and population genotype data from public databases were reviewed, covering 94 Colombian adults genotyped under the CLM (Colombians from Medellín) reference. Data traceability was ensured through ID registration in the 1000 Genomes project database, guaranteeing informed consent and bioethical approval. Eleven genes *(GSTP1, ATP7B, BDNF, GCLC, GCLM, MT1A, MT4, ABCC2, ABCB1* , *GPX1* , and *GPX4)* with 18 polymorphisms distributed across ten chromosomes were analyzed using the SNPstats^TM^ program. The X^2^ test was applied to evaluate the Hardy-Weinberg equilibrium, considering deviations with p < 0.05 as significant.

**Results.:**

The results showed a high probability of an association between neurodegenerative diseases such as Alzheimer's and Parkinson's and mercury exposure in individuals with genetic variants related to glutathione metabolism and mercury transport and excretion pathways.

**Conclusions.:**

Genetic alterations or their expression involving mercury bioaccumulation, its crossing of the blood-brain barrier, central nervous system inflammation, and oxidative stress from reactive oxygen and nitrogen species increase the risk of developing Alzheimer's and Parkinson's disease.

Las enfermedades neurodegenerativas, como la de Alzheimer y la de Parkinson, se derivan de anomalías en el metabolismo de moléculas esenciales del ciclo celular ^1^. En sus etapas iniciales, el aumento de las especies reactivas de oxígeno y de nitrógeno provoca un estrés oxidativo que altera el metabolismo del glutatión (GSH), el cual, junto con otros antioxidantes, contribuye a neutralizar el daño oxidativo y a mantener el correcto funcionamiento del sistema nervioso central ^2^. Además de estos mecanismos endógenos, existe evidencia de que la interacción entre la predisposición genética y la exposición a tóxicos ambientales, desempeña un papel fundamental en el desarrollo de trastornos del neurodesarrollo y en procesos neurotóxicos ^3^. Esto sugiere que una mayor susceptibilidad genética podría incrementar el riesgo de enfermedades neurodegenerativas en poblaciones expuestas a metales pesados, como el mercurio (Hg), dada su interferencia directa en el metabolismo del glutatión ^4^.

En Colombia, en un estudio liderado por el Ministerio de Salud y Protección Social ^5^, se determinó que las principales fuentes de liberación de mercurio se ubican donde se adelantan actividades de minería de oro a pequeña y mediana escala. La concentración del metal en sitios como el departamento de Medellín -y su subregión del Bajo Cauca-, resalta la necesidad de investigar los efectos de esta exposición sobre la salud humana y el medio ambiente.

El mercurio es un metal pesado que se considera una neurotoxina, con afinidad por los tejidos grasos. Se acumula principalmente en los riñones y el cerebro, y presenta una gran toxicidad ^6^. Este elemento forma enlaces químicos con el glutatión mediante la cisteína, lo que permite su eliminación por la orina o las heces gracias a las propiedades antioxidantes y desintoxicantes del glutatión ^7^. No obstante, los errores en sus rutas metabólicas pueden tener implicaciones importantes en el desarrollo de enfermedades neurodegenerativas ^8^.

En la revisión bibliográfica, se encontraron, al menos, once genes correlacionados con diferentes reacciones a la exposición y el metabolismo del mercurio en humanos: *GSTP1, MT4, ATP7B, BDNF*(6,9), *GCLC, GCLM* (3,6,9), *MT1A, ABCC2 (MRP2), ABCB1 (MDR1), GPX1* y *GPX4* (6). Se ha sugerido que estos genes y sus variantes podrían explicar la relación entre la exposición al mercurio y el desarrollo de enfermedades neurodegenerativas, dependiendo del grado de predisposición genética. Por esta razón, dichos genes constituyen el foco de interés de este estudio.

El gen *GSTP1* codifica la enzima GSTP1 (glutatión S-transferasa pi 1), la cual participa en la desintoxicación de los compuestos xenobióticos mediante la conjugación nucleofílica del glutatión reducido (GSH: γ-Glu-Cys-Gly) con centros electrófilos presentes en múltiples estructuras tóxicas, incluido el mercurio (10). Esta actividad catalítica es esencial para la neutralización y la eliminación del mercurio del organismo.

El polimorfismo Ile105Val (rs1695) genera una variante en la que la capacidad enzimática disminuye y la susceptibilidad a la inhibición por Hg2^+^ y metilmercurio (CH_3_Hg+) aumenta, lo cual se ha asociado con mayores concentraciones de mercurio en los tejidos y deterioro neurocognitivo en las poblaciones expuestas ^9^. Por otra parte, la variante Ala114Val (rs1138272) también compromete la eficiencia y la sensibilidad de la enzima GSTP1, lo cual afecta su desempeño como desintoxicante ^6^. En conjunto, estos polimorfismos -rs1695 y rs1138272- actúan como moduladores genéticos fundamentales en la capacidad de desintoxicación contra el mercurio mediada por la ruta del glutatión y podrían explicar la variabilidad individual en la susceptibilidad a los efectos tóxicos del metal.

Por otro lado, el gen *GCLC* codifica la subunidad catalítica de la enzima GCL (glutamato cisteína ligasa), responsable de limitar la síntesis del glutatión, un antioxidante esencial en la desintoxicación de metales pesados como el mercurio ^3^. En este gen, el polimorfismo rs1555903 se ha identificado como un factor genético relevante en la acumulación de mercurio, particularmente en matrices biológicas como cabello, sangre y orina. En un estudio previo, se observó que el alelo C de rs1555903 -considerado menos frecuente- presentaba una asociación significativa con mayores concentraciones de mercurio en las tres matrices mencionadas, incluso después de la corrección por Bonferroni ^10^. Este hallazgo sugiere que dicha variante podría reducir la eficiencia de síntesis de glutatión, disminuyendo la capacidad de neutralización del mercurio en el organismo. Además, en estudios independientes se ha vinculado al polimorfismo SNP rs17883901, con una menor actividad promotora del gen *GCLC,* lo cual también afecta la expresión génica y la disponibilidad de glutatión en casos de exposición a metilmercurio ^6^^,^^9^.

De forma complementaria, el gen *GCLM* codifica la subunidad modificadora de la misma enzima (glutamato cisteína ligasa o GCL), la cual participa en la regulación de la actividad catalítica de la GCLC; en conjunto la GCLM y la GCLC conforman el sistema enzimático responsable de la síntesis del glutatión (GSH), un proceso esencial en la desintoxicación del mercurio. En este caso, la variante rs41303970, ubicada en la región promotora del gen *GCLM,* se ha relacionado con una menor expresión del gen y una reducción en la capacidad de reacción frente al estrés oxidativo inducido por el mercurio, especialmente en las muestras de cabello ^3^^,^^9^. En conjunto, las variantes funcionales en el *GCLC* y el *GCLM* influyen directamente en las concentraciones corporales de mercurio y podrían considerarse biomarcadores de susceptibilidad genética ante la exposición crónica a este metal.

Los genes *GPX1* y *GPX4* codifican las enzimas glutatión peroxidasa citosólica y glutatión peroxidasa mitocondrial, cuya función principal es reducir peróxidos de hidrógeno y lípidos a productos no tóxicos, utilizando el glutatión reducido como cofactor. Esta actividad es fundamental para la defensa celular frente al estrés oxidativo, incluido aquel que se genera por la exposición a metales pesados como el mercurio ^6^.

En el caso del *GPX1* , el polimorfismo rs1050450 (Pro198Leu) puede reducir su actividad enzimática y alterar su localización subcelular. Aunque los estudios en poblaciones expuestas han mostrado resultados inconsistentes en cuanto a la acumulación de mercurio, las investigaciones experimentales respaldan que esta variante compromete la función antioxidante, especialmente en condiciones de deficiencia de selenio ^6^^,^^9^. Por su parte, las variantes 718T y TT del gen *GPX4* se han asociado con bajas concentraciones de mercurio, lo que sugiere un posible efecto protector frente a enfermedades neurológicas ^11^.

Por otro lado, el SNP rs713041, localizado en la región 3'UTR del *GPX4,* podría afectar la estabilidad del ARN mensajero o la eficiencia de traducción de este gen. Este polimorfismo ha sido mencionado en revisiones sobre la toxicidad del mercurio en relación con el *GPX1* y el *GPX4.* Aunque hasta ahora no se han reportado estudios epidemiológicos directos en que se evalúe la relación entre rs713041 y los niveles de mercurio, su posible función reguladora lo convierte en un candidato relevante para explicar la variabilidad de los efectos neurotóxicos del mercurio ^11^.

Los genes *MT1A* y *MT4* codifican dos isoformas de la familia de las metalotioneínas, proteínas ricas en cisteína ^11^. Estas isoformas tienen una gran afinidad por metales pesados como el mercurio, lo que les confiere propiedades desintoxicantes y antioxidantes esenciales para la defensa celular, principalmente frente a este tipo de metales ^12^. En el gen *MT1A* se han identificado los polimorfismos rs8052394 (51Lys>Arg) y rs11640851 (27Thr>Asn), mientras que, en el MT4, se ha descrito la variante rs11643815 (48Gly>Asp). Estas variantes pueden modificar tanto la actividad de transcripción como la conformación estructural de las proteínas, afectando potencialmente su capacidad funcional frente al estrés oxidativo y la exposición a metales pesados ^6^.

Ambas isoformas *MT1A* y *MT4* participan en la modulación de la inflamación inducida por especies reactivas de oxígeno y nitrógeno, lo que evidencia un papel esencial en la neuroprotección. En estudios experimentales, se ha demostrado que la sobreexpresión de los genes de metalotioneína atenúa los procesos neurodegenerativos, como los observados en la enfermedad de Parkinson, al reducir la formación de especies reactivas de oxígeno y nitrógeno. Por el contrario, una disminución de su expresión se asocia con un aumento del daño neuronal, lo que resalta el papel protector de estas proteínas en el sistema nervioso central.

Por su parte, el *ATP7B* es un gen que codifica una ATPasa de tipo P, principalmente involucrada en el transporte de cobre a través de membranas celulares ^13^. No obstante, como lo señalan Andreoli y Sprovieri ^6^, variantes del *ATP7B,* como los polimorfismos rs1061472 (832Lys>Arg) y rs732774 (952Arg>Lys), podrían estar implicadas también en el transporte de otros metales, incluido el mercurio. En línea con esto, Parajuli *et al.*^9^ demostraron que estos polimorfismos se asocian con menores concentraciones de mercurio en el cabello humano, lo que sugiere que el *ATP7B* podría participar en la modulación de los niveles sistémicos de mercurio, posiblemente actuando como transportador de este metal en humanos.

El gen *BDNF* codifica un factor neurotrófico derivado del cerebro y participa en la supervivencia neuronal y la plasticidad sináptica ^14^. Su expresión está estrechamente relacionada con el mantenimiento funcional del sistema nervioso central. Según Andreoli y Sprovieri ^6^, el polimorfismo rs6265 (66Val > Met) en el *BDNF* podría afectar la viabilidad de las neuronas de las estrías y se ha asociado con una mayor susceptibilidad a la neurotoxicidad inducida por mercurio.

Parajuli *et al.*^9^ también identificaron una relación entre esta misma variante (rs6265) y escasas concentraciones de mercurio en el cabello, lo que sugiere un posible papel modulador de *BDNF* en la toxicocinética del metal. Esto refuerza la hipótesis de que el *BDNF* no solo cumple funciones neurotróficas, sino que también podría intervenir en mecanismos de protección frente al daño neurotóxico por exposición al mercurio ^6^. Además, como destacan Villa-Cedillo y Saucedo-Cárdenas ^15^, el BDNF *(Brain-Derived Neurotrophic Factor)* se considera una molécula clave en enfermedades neurodegenerativas como la de Alzheimer, debido a que el hipocampo -una de las regiones más afectadas en esta enfermedad- depende en gran medida de su señalización para mantener la integridad sináptica y neuronal.

Los genes *ABCC2* y *ABCB1* codifican proteínas transportadoras pertenecientes a la familia de casetes de unión a ATP, ABC *(ATP-Binding Cassette),* las cuales desempeñan un papel clave en la defensa del organismo contra sustancias tóxicas lipofílicas, como el mercurio, especialmente aquellas que pueden atravesar la barrera hematoencefálica ^16^.

Por su parte, el gen *ABCB1* codifica la glucoproteína P, una proteína transportadora de tipo ABC que extrae compuestos endógenos y exógenos del sistema nervioso central hacia la circulación sanguínea, contribuyendo a la protección del encéfalo frente a agentes neurotóxicos ^17^. Se ha propuesto que ciertas variantes genéticas del *ABCB1* están relacionadas con el riesgo de desarrollar enfermedades neurodegenerativas como la de Parkinson, la de Alzheimer y la esclerosis múltiple, debido a su papel en los procesos inflamatorios y en la protección de la mielina. En este contexto, el alelo G2677 se ha asociado con mayor susceptibilidad, mientras que los genotipos T3435 y CT3435 podrían ejercer un efecto protector ^17^.

El gen *ABCC2* codifica la proteína de resistencia a múltiples fármacos de tipo 2, actuando como una bomba de eflujo responsable del transporte activo de toxinas y xenobióticos hacia el exterior de órganos como el hígado, los riñones, el intestino y la placenta, facilitando su excreción. Según Grisolía ^8^, el *ABCC2* participa en el transporte y la eliminación del mercurio, función que puede estar modulada por polimorfismos como rs1885301 y rs717620 en la región 5'UTR, así como por el polimorfismo codificante rs2273697 (417Val>Ile). De manera similar, el *ABCB1* también interviene en la expulsión de mercurio, influido por variantes como rs2032582 (893Ala>Ser) y rs1202169 en la región 3'UTR.

Por lo anterior, este estudio pretende identificar y analizar alelos y genes relacionados con enfermedades neurodegenerativas en personas expuestas al mercurio en Colombia. El análisis se basa en la determinación de frecuencias alélicas, haplotípicas y polimorfismos individuales en genes implicados en el metabolismo del glutatión, el transporte y la excreción del mercurio.

## Materiales y métodos

### Tipo de estudio

Este estudio corresponde a una investigación genética inicial, de tipo no experimental y descriptivo, con un enfoque epidemiológico genético. Su objetivo fue identificar y analizar las frecuencias de alelos y genes asociados a enfermedades neurodegenerativas en relación con la exposición a metales pesados como el mercurio en la población de Medellín. La metodología se basó en la revisión de la literatura científica y en el análisis de datos genómicos provenientes de bases públicas de datos, lo cual permite proponer candidatos genéticos y establecer una base para estudios posteriores de asociación genotipo-fenotipo en contextos de mezcla poblacional.

### Población y muestra

La muestra estuvo conformada por 94 adultos colombianos de Medellín (Antioquia), genotipificados bajo la referencia poblacional CLM *(Colombians from Medellín)* del *Proyecto 1000 Genomas.* Esta población fue seleccionada por su reconocida diversidad genética ancestral y por la disponibilidad pública de los datos. La muestra incluyó 51 mujeres y 43 hombres. Por razones de confidencialidad, no se dispone de información sobre la edad de los participantes. La trazabilidad de los datos está garantizada mediante su registro individual en la base de datos del *Proyecto 1000 Genomas,* cumpliendo con los requisitos éticos y de consentimiento informado.

### Recolección de datos y búsqueda bibliográfica

Se realizó una revisión exhaustiva de literatura científica para identificar genes y variantes asociadas con enfermedades neurodegenerativas vinculadas a la exposición al mercurio. La búsqueda abarcó estudios poblacionales previos y reportes experimentales que incluyeran asociaciones genéticas con la toxicocinética del mercurio. Además, se consultaron múltiples bases de datos genéticas especializadas, tales como: OMIM *(Online Mendelian Inheritance in Man);* ClinVar; GWAS *(Genome-Wide Association Studies)* Central y Catalog; y NIMH *(National Institute of Mental Health).* Se extrajeron las variantes alélicas relevantes, así como sus funciones biológicas, productos génicos asociados y localización cromosómica.

La estrategia de búsqueda bibliográfica incluyó el uso de palabras clave y operadores booleanos combinados, tales como: ("mercury" OR "Hg") AND ("genetic polymorphism" OR "SNP") AND ("neurodegenerative disease" OR "Parkinson" OR "Alzheimer") AND ("glutathione" OR "GSH") AND ("toxicity" OR "exposure"). Las búsquedas se realizaron en inglés y español, y se limitaron a publicaciones científicas revisadas por pares.

Se consideró un rango temporal de los últimos 26 años (1999-2025) para asegurar la inclusión de estudios relevantes y actualizados. Los criterios de inclusión fueron: artículos originales o revisiones sistemáticas que reportaran asociaciones entre polimorfismos genéticos y exposición al mercurio en humanos, con énfasis en genes implicados en el metabolismo de xenobióticos, estrés oxidativo, neuroprotección o transporte de metales. Se excluyeron las publicaciones duplicadas, los estudios con animales sin evidencia equivalente en humanos, los artículos sin acceso al texto completo y los estudios sin análisis genético. Esta estrategia permitió construir una base sólida para la selección de genes candidato y sus variantes funcionales, priorizando aquellas con relevancia biomédica demostrada y aplicabilidad en el contexto poblacional colombiano.

### Obtención de los datos genómicos

Los datos genómicos empleados en este estudio provienen del repositorio internacional IGSR *(International Genome Sample Resource),* que alberga las muestras del proyecto 1000 Genomas, una iniciativa internacional que tiene como objetivo mapear la variabilidad genética de la población humana mediante el análisis de polimorfismos de un solo nucleótido en distintas poblaciones globales.

En el contexto de este estudio, se utilizaron los datos correspondientes a individuos colombianos de Medellín, codificados bajo la referencia CLM *(Colombians from Medellín).* Esta cohorte fue seleccionada por su disponibilidad abierta, su validación ética y por representar de manera significativa la diversidad genética ancestral de la región. La información genética de estos individuos incluye perfiles de variantes genómicas individuales, los cuales fueron genotipificados bajo condiciones estandarizadas y con trazabilidad garantizada mediante identificadores únicos.

La inclusión de esta población ha sido clave para diversas investigaciones sobre evolución humana, genética de poblaciones y enfermedades complejas. En el presente estudio, estos datos constituyen la base para el análisis de frecuencias alélicas, genotípicas y haplotípicas de variantes previamente identificadas como relevantes en la toxicodinámica del mercurio y en la susceptibilidad a enfermedades neurodegenerativas.

### Análisis de los datos

Las frecuencias alélicas, genotípicas, haplotípicas y de combinaciones alélicas intercromosómicas, fueron calculadas a partir de los datos crudos correspondientes a la población CLM disponibles en la base de datos del *Proyecto 1000 Genomas.* Para el análisis estadístico, se utilizó el programa SNPstats™, una herramienta gratuita basada en el lenguaje de programación R.

Con el fin de garantizar la calidad de los perfiles genéticos analizados, se aplicó la prueba de X^2^ para evaluar el equilibrio de Hardy-Weinberg a partir de las distribuciones genotípicas observadas. Esta prueba, de uso estándar en genética de poblaciones, permite identificar desviaciones atribuibles a errores de genotipificación. Solo se consideraron significativas aquellas desviaciones con un valor de p menor de 0,05, siguiendo el criterio propuesto por Mayo ^18^.

### Construcción de redes génicas

Para explorar las posibles interacciones funcionales entre los polimorfismos evaluados en los genes *GSTP1, GCLC, GCLM, GPX1, GPX4, MT1A, MT4, ATP7B, BDNF, ABCC2* y *ABCB1,* se construyó una red de interacción génica utilizando el programa GeneMANIA, accesible desde la plataforma NDEx. Esta herramienta permite integrar datos biológicos de múltiples fuentes para predecir relaciones funcionales entre genes, facilitando la priorización de funciones génicas relevantes. Se ingresaron como genes de consulta aquellos correspondientes a los polimorfismos incluidos en este estudio y se permitió la incorporación automática de genes funcionalmente relacionados, basada en el algoritmo predictivo del programa utilizado. La metodología se basa en el enfoque publicado por Warde-Farley *et al.*^19^.

## Resultados

### Descripción de la muestra del estudio

La muestra analizada estuvo compuesta por 94 adultos colombianos provenientes de Medellín (Antioquia), de los cuales 51 eran mujeres y 43 hombres. A todos se les realizó la genotipificación para 18 polimorfismos de interés por su relevancia en el metabolismo del mercurio y en las enfermedades neurodegenerativas. Los datos genéticos, obtenidos del *Proyecto 1000 Genomas,* mostraron una gran calidad, con cumplimiento del equilibrio de Hardy-Weinberg en 17 de los 18 *loci* analizados.

El único marcador que presentó una desviación significativa fue rs12076499 (p = 0,016), por lo cual fue excluido de los análisis posteriores. Este hallazgo representa un índice de genotipificación exitosa del 94,4 %, y respalda la integridad y adecuación de los datos para los análisis de las frecuencias alélicas y haplotípicas. Los valores de p del equilibrio de Hardy-Weinberg, así como las frecuencias alélicas y genotípicas de todos los 18 polimorfismos estudiados, se encuentran detallados en el cuadro 1.

**Cuadro 1:** Frecuencias alélicas y genotípicas de los polimorfismos estudiados*

***GSTP1* rs1695**							***ATP7B* rs1061472**					
Alelo	Número	Proporción	Genotipo	Número	Proporción		Alelo	Número	Proporción	Genotipo	Número	Proporción
A	121	0,64	A/A	36	0,38		C	95	0,51	C/C	22	0,23
G	67	0,36	A/G	49	0,52		T	93	0,49	C/T	51	0,54
EHW = 0,26			G/G	9	0,1		EHW = 0,54			T/T	21	0,22
*GSTP1* rs1138272							*ATP7B* rs732774					
Alelo	Número	Proporción	Genotipo	Número	Proporción		Alelo	Número	Proporción	Genotipo	Número	Proporción
C	183	0,97	C/C	89	0,95		T	114	0,61	C/C	14	0,15
T	5	0,03	C/T	5	0,05		C	74	0,39	T/C	46	0,49
EHW = 1							EHW = 1			T/T	34	0,36
***GCLC* rs17883901**							***BDNF* rs6265**					
Alelo	Número	Proporción	Genotipo	Número	Proporción		Alelo	Número	Proporción	Genotipo	Número	Proporción
G	178	0,95	G/A	10	0,11		C	158	0,84	C/C	65	0,69
A	10	0,05	G/G	84	0,89		T	30	0,16	C/T	28	0,3
EHW = 1							EHW = 0,45			T/T	1	0,01
***GCLC* rs1555903**							***ABCC2* rs1885301**					
Alelo	Número	Proporción	Genotipo	Número	Proporción		Alelo	Número	Proporción	Genotipo	Número	Proporción
T	131	0,7	C/C	10	0,11		G	105	0,56	A/A	19	0,2
C	57	0,3	T/C	37	0,39		A	83	0,44	G/A	45	0,48
EHW = 0,47			T/T	47	0,5		EHW = 0,83			G/G	30	0,32
***GCLM* rs41303970**							***ABCC2* rs717620**					
Alelo	Número	Proporción	Genotipo	Número	Proporción		Alelo	Número	Proporción	Genotipo	Número	Proporción
G	146	0,78	A/A	4	0,04		C	154	0,82	C/C	62	0,66
A	42	0,22	G/A	34	0,36		T	34	0,18	C/T	30	0,32
EHW = 1			G/G	56	0,6		EHW = 0,73			T/T	2	0,02
***GPX4* rs713041**							***ABCC2* rs2273697**					
Alelo	Número	Proporción	Genotipo	Número	Proporción		Alelo	Número	Proporción	Genotipo	Número	Proporción
C	121	0,64	C/C	38	0,4		G	159	0,85	A/A	2	0,02
T	67	0,36	C/T	45	0,48		A	29	0,15	G/A	25	0,27
EHW= 0,82		T/T	11	0,12		EHW = 1				G/G	67	0,71
***MT1A* rs8052394**							***ABCB1* rs12076499**					
Alelo	Número	Proporción	Genotipo	Número	Proporción		Alelo	Número	Proporción	Genotipo	Número	Proporción
A	155	0,82	A/A	63	0,67		C	185	0,98	C/C	92	0,98
G	33	0,18	A/G	29	0,31		T	3	0,02	C/T	1	0,01
EHW = 0,73			G/G	2	0,02		EHW = 0,016			T/T	1	0,01
***MT1A* rs11640851**							***ABCB1* rs1202169**					
Alelo	Número	Proporción	Genotipo	Número	Proporción		Alelo	Número	Proporción	Genotipo	Número	Proporción
A	110	0,59	A/A	35	0,37		T	108	0,57	C/C	20	0,21
C	78	0,41	A/C	40	0,43		C	80	0,43	T/C	40	0,43
EHW = 0,29			C/C	19	0,2		EHW = 0,21			T/T	34	0,36
***MT4* rs11643815**							***GPX1* rs1050450**					
Alelo	Número	Proporción	Genotipo	Número	Proporción		Alelo	Número	Proporción	Genotipo	Número	Proporción
G	176	0,94	A/A	1	0,01		G	151	0,8	A/A	3	0,03
A	12	0,06	G/A	10	0,11		A	37	0,2	G/A	31	0,33
EHW = 0,31		G/G	83	0,88		EHW = 1				G/G	60	0,64

EHW: equilibrio de Hardy Weinberg

* Se presentan las frecuencias alélicas y genotípicas calculadas para los polimorfismos genéticos usando el programa SNPStats^TM^ (2024).

### Frecuencias alélicas y genotípicas

Los polimorfismos evaluados corresponden a genes clave implicados en el metabolismo del glutatión, la reacción antioxidante y el transporte de metales pesados, incluyendo *GSTP1, GCLC, GCLM, GPX1, GPX4, MT1A, MT4, ATP7B, BDNF, ABCC2* y *ABCB1* .

En términos generales, se observó una heterogeneidad moderada en las frecuencias alélicas entre los distintos *loci.* Algunos polimorfismos, como rs1695 *(GSTP1)* y rs1061472 *(ATP7B),* presentaron alelos distribuidos de forma relativamente equilibrada (A = 0,64 / G = 0,36 y C = 0,51 / T = 0,49, respectivamente), sin predominancia extrema de un solo alelo, lo que sugiere una estructura genética estable para estos marcadores en la población analizada. Sin embargo, otros locus mostraron amplias diferencias en la distribución de alelos, como el rs11643815 *(MT4),* donde el alelo G fue mayoritario (94 %) frente al alelo A (6 %).

En el extremo opuesto, se identificaron alelos minoritarios con proporciones particularmente bajas, como el alelo T del rs12076499 *(ABCB1),* con una frecuencia del 2 %. Este marcador también mostró una desviación significativa del equilibrio de Hardy-Weinberg (p = 0,016), por lo que se excluyó de los análisis posteriores, como se mencionó en la sección de metodología.

Estas variaciones reflejan la diversidad genética interindividual en la muestra estudiada y, al mismo tiempo, validan la calidad de los datos obtenidos, dado que 17 de los 18 polimorfismos cumplieron con los criterios esperados de equilibrio de Hardy-Weinberg. Este perfil proporciona una base sólida para los análisis posteriores de haplotipos y combinaciones alélicas, los cuales se detallan en la siguiente sección.

### Frecuencias de haplotipos y combinaciones alélicas

Las frecuencias de haplotipos y combinaciones alélicas obtenidas a partir de los 17 polimorfismos analizados se presentan en el cuadro 2. Se diferenciaron dos enfoques complementarios: el análisis de haplotipos (combinaciones de alelos ubicados en un mismo cromosoma) y de combinaciones alélicas (agrupaciones funcionales entre variantes localizadas en distintos cromosomas).

**Cuadro 2:**
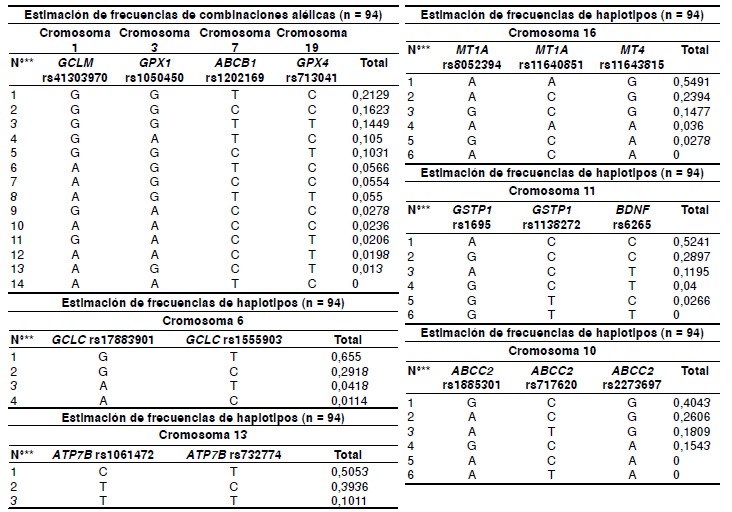
Frecuencias de haplotipos y combinaciones alélicas (n = 94)*

* Las frecuencias de haplotipos y combinaciones alélicas fueron calculadas a partir de los genotipos en el programa SNPStats^TM^ results (2024).

** Número del haplotipo o combinación alélica

Se identificaron bloques haplotípicos en los cromosomas 6, 10, 11, 13 y 16, formados por polimorfismos físicamente cercanos. En el cromosoma 6 (rs17883901-rs1555903), el haplotipo más frecuente fue G-T, con una proporción de 0,655, seguido por G-C (0,2918) y A-T (0,0418). Este patrón sugiere una fuerte asociación entre estos locus, lo que es compatible con un desequilibrio de ligamiento.

En el cromosoma 11, el haplotipo A-C-C, conformado por variantes en *GSTP1* y *BDNF,* fue el más prevalente (0,5241), seguido por el G-C-C (0,2897) y el A-C-T (0,1195).

En los cromosomas 10, 13 y 16, también se observaron múltiples haplotipos con frecuencias intermedias y dispersas. Por ejemplo, en el cromosoma 16, el haplotipo A-A-G (rs8052394-rs11640851-rs11643815) presentó una frecuencia de 0,5491, seguido por A-C-G (0,2394) y otras combinaciones minoritarias. Estas estructuras reflejan bloques de herencia conjunta en regiones específicas del genoma y ofrecen información sobre la organización genética local en la población estudiada.

Asimismo, se evaluaron combinaciones alélicas entre variantes ubicadas en distintos cromosomas, con potencial relevancia funcional para los procesos de desintoxicación y reacción antioxidante. Este análisis incluyó los polimorfismos rs41303970 *(GCLM,* cromosoma 1), rs1050450 *(GPX1,* cromosoma 3), rs1202169 *(ABCB1,* cromosoma 7) y rs713041 *(GPX4,* cromosoma 19). Estas combinaciones no representan haplotipos en sentido genético, pero fueron analizadas por su interés biológico. La combinación G-G-T-C fue la más frecuente (0,2129), seguida por G-G-C-C (0,1623) y G-G-T-T (0,1449). Las demás combinaciones presentaron frecuencias más bajas, inferiores al 10 %. Este conjunto de resultados proporciona una base sólida para el análisis funcional de las interacciones genéticas mediante redes de interacción biológica, desarrollado en la siguiente sección.

### Redes génicas

La red generada integra múltiples fuentes de evidencia funcional: coexpresión génica, interacción física directa, colocalización subcelular, participación en rutas metabólicas compartidas, interacción genética y presencia de dominios proteicos comunes. Este enfoque permitió construir un mapa funcional de relaciones potenciales entre los genes implicados en la desintoxicación de metales pesados, transporte de xenobióticos y reacción antioxidante.

**Figura 1:**
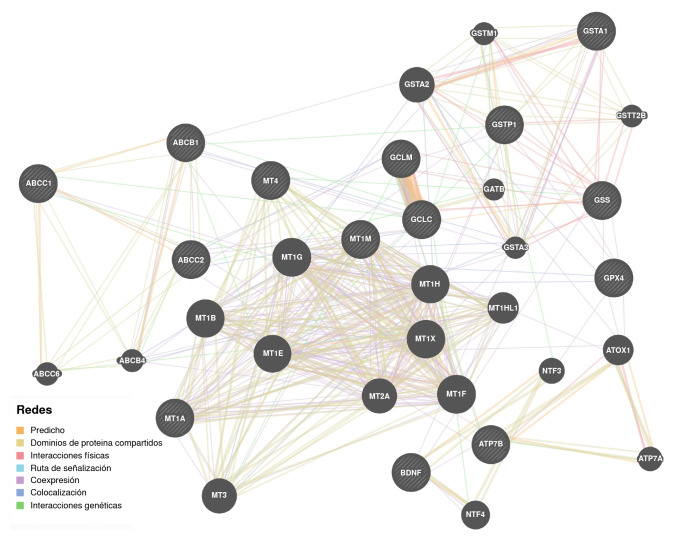
Red génica construida con los genes *GSTP1, MT4, ATP7B, BDNF, GCLC, GCLM, MT1A, ABCC2, ABCB1, GPX4*

En la figura 1, se observa una red densamente conectada, en la que se destacan genes centrales *(hubs)* como *GSTP1, GCLM* y *GCLC,* por su elevado número de interacciones funcionales. Estos genes aparecen con genes centrales de mayor tamaño, lo que refleja su alto grado de conectividad y sugiere un papel integrador fundamental en los procesos celulares analizados. Aunque *MT1A* está presente en la red, su conectividad es más limitada, por lo que no se clasifica como un gen central aunque sigue siendo funcionalmente relevante en la desintoxicación metálica.

Las aristas *(edges)* entre genes varían en grosor, de acuerdo con el peso asignado por el modelo de predicción: las aristas más gruesas representan interacciones más fuertes o recurrentes entre los genes centrales conectados. Asimismo, el color de las aristas distingue el tipo de evidencia funcional; por ejemplo, la coexpresión se representa en color lila, las interacciones físicas en rojo y las genéticas en verde. Un patrón notable de coexpresión puede observarse entre los genes del sistema de glutatión, como GSS, *GCLC, GSTA3, GSTA1, GSTT2B* y *GSTP1,* lo cual indica una participación coordinada en mecanismos antioxidantes celulares.

En conjunto, esta red permite visualizar el contexto funcional ampliado de los genes evaluados en este estudio, proporcionando una base para interpretar sus posibles interacciones biológicas y su rol en la susceptibilidad genética a los efectos tóxicos del mercurio.

## Discusión

En este trabajo se caracterizan por primera vez, en una cohorte colombiana, las variantes de los genes que regulan las principales rutas de desintoxicación y transporte del mercurio y su posible vínculo con la susceptibilidad a enfermedades neurodegenerativas. Para ello, se analizaron 94 adultos sanos (51 mujeres y 43 hombres) del subgrupo CLM del *Proyecto 1000 Genomas,* cuya probada mezcla ancestral europea, africana e indígena ^20^^,^^21^ ofrece una situación genética singular en Latinoamérica, históricamente poco representada en la literatura genómica. Aunque el tamaño muestral es moderado, la rica heterogeneidad genética de esta población proporciona un modelo de gran resolución para identificar variantes circulantes relevantes y explorar cómo podrían modular el riesgo neurodegenerativo en contextos de exposición ambiental al mercurio propios de Colombia.

Se genotipificaron 18 polimorfismos de un solo nucleótido repartidos en 11 genes que intervienen en la respuesta celular al mercurio: *GCLC* y *GCLM* (biosíntesis y reciclaje de glutatión); *GSTP1, GPX1* y *GPX4* (conjugación y defensa antioxidante); *MT1A* y *MT4* (quelación de metales); *ABCB1, ABCC2* y *ATP7B* (transporte y excreción biliar); y *BDNF* (señalización neurotrófica). La solidez técnica quedó patente al observarse equilibrio de Hardy-Weinberg en 17 de los 18 marcadores (p > 0,05), descartándose errores sistemáticos de laboratorio. La única excepción, *ABCB1* rs12076499 (p = 0,016), se mantuvo tras réplicas concordantes, lo que sugiere que, en lugar de una falla metodológica, se trató de un posible fenómeno biológico de selección, deriva genética o efecto fundador.

A pesar de las limitaciones de los datos utilizados, este panel genómico ofrece la solidez necesaria para estimar frecuencias alélicas, reconstruir haplotipos y modelar el desequilibrio de ligamiento, estableciendo una base confiable para indagar cómo esta variabilidad modula la susceptibilidad a la neurotoxicidad por mercurio en la población CLM.

Desde la óptica de la genética epidemiológica, esta cohorte se establece como un referente local al describir por primera vez las frecuencias alélicas y genotípicas, las combinaciones intercromosómicas y los haplotipos en bloques de ligamiento de once genes que orquestan la desintoxicación y el transporte de mercurio. La comparación con bases de datos globales revela perfiles de riesgo distintivos, permite inferir posibles adaptaciones o presiones selectivas ancestrales, y destaca la gran prevalencia de alelos, genotipos y haplotipos que debilitan la capacidad antioxidante, un rasgo que perfila subgrupos especialmente vulnerables a la exposición crónica al mercurio. Estos hallazgos abren camino, por un lado, a estudios de asociación de mayor escala, preferentemente longitudinales, que integren biomonitoreo de mercurio, evaluaciones neurocognitivas y un registro exhaustivo de variables ambientales y clínicas. Por otro lado, marcan un precedente para la ampliación de la muestra en futuras investigaciones.

El análisis de las frecuencias alélicas, genotípicas y haplotípicas refuerza la hipótesis de una vulnerabilidad genética latente en la población estudiada. Según las estimaciones de haplotipos (cuadro 2), la enzima GCLC -codificada en el cromosoma 6- resulta de interés por los polimorfismos rs17883901 y rs1555903. El haplotipo GT presenta una frecuencia alélica del 65 % (0,655); así pues, en una cohorte de 100 individuos (200 cromosomas), 130 genomas presentarían mayor probabilidad de asociarse con enfermedades neurodegenerativas como la de Parkinson y la de Alzheimer ^2^, ya que estos polimorfismos limitan la velocidad de síntesis de glutatión ^3^.

La disminución de glutatión incrementa las especies reactivas de oxígeno y nitrógeno, provocando daño oxidativo e inflamatorio en el sistema nervioso central ^2^. En particular, el alelo G de rs17883901 se ha vinculado con un mayor riesgo de acumulación de mercurio, mientras que la variante A parece ejercer un efecto protector, asociado a concentraciones bajas de este metal en la población china ^22^. De manera similar, en nuestra muestra no se detectó el genotipo homocigoto protector AA y se observó una gran frecuencia del alelo de riesgo G (cuadro 1).

Por su parte, el alelo C de rs1555903 se ha relacionado con retención de metilmercurio en la sangre del cordón umbilical en niños de Inglaterra. En la población colombiana, aunque no se asoció con concentraciones sanguíneas de mercurio, sí se vinculó con lesión renal: portadores del alelo C presentaron una tasa de filtración glomerular estimada más baja ^23^. Finalmente, el grupo de mayor riesgo en nuestra cohorte corresponde al haplotipo 2 (GC), presente en el 29 % de los individuos, portadores simultáneos de ambos alelos de riesgo.

Las metalotioneínas codificadas en el cromosoma 16 constituyen el segundo bloque de mayor interés. El haplotipo integrado por *MT1A* rs8052394 A, *MT1A* rs11640851 A y *MT4* rs11643815 G (AAG), muestra una frecuencia estimada del 55 % (0,549), lo que implica que en una población hipotética de 100 individuos (200 cromosomas), alrededor de 108 genomas portarían dicha combinación. Se ha descrito que estos alelos modifican la actividad transcripcional y la conformación de proteínas con función neuroprotectora al fijar el mercurio, favoreciendo procesos neurodegenerativos como la enfermedad de Parkinson ^11^^,^^14^.

Dentro de este bloque, el alelo G de *MT4* rs11643815 -presente en el 94 % de los genotipos de la muestra y componente de los haplotipos 1, 2 y 3- se considera un factor de riesgo toxicocinético, ya que se asocia con mayores cargas corporales de mercurio en las poblaciones expuestas ^3^^,^^6^. El cambio p.Gly48Asp que introduce esta variante reduce la afinidad de los residuos de cisteína por Hg^2+^, limita la quelación intracelular y entorpece la exportación Zn^2+^/Hg^2+^ hacia los queratinocitos, propiciando su retención sistémica.

El alelo A de *MT1A* rs8052394 se ha vinculado a concentraciones elevadas de mercurio capilar cuando el consumo de pescado es alto ^24^. Además, los genotipos G-G o G-A de este marcador duplican el riesgo de deterioro cognitivo leve en individuos del tercil superior de mercurio sanguíneo ^25^. La sustitución Lys→Arg que provoca rs8052394, degrada el bolsillo de unión a cationes divalentes, disminuye la afinidad y la velocidad de exportación de complejos Hg-MT y, en consecuencia, potencia bioacumulación, estrés oxidativo y neuroinflamación.

El haplotipo 3 (G-C-G) sobresale como el de mayor riesgo, ya que combina los alelos anteriores con la variante C de *MT1A* rs11640851. Este alelo contrasta con su contraparte A, relacionada con menores concentraciones plasmáticas y capilares de mercurio y con biomarcadores reducidos de estrés oxidativo en poblaciones de la amazonía brasileña ^26^ y de los Estados Unidos ^27^, lo que sugiere un efecto protector frente a la acumulación de mercurio.

En el cromosoma 11, la enzima GSTP1 (polimorfismos rs1695 y rs1138272) y el BDNF (polimorfismo rs6265) conforman un haplotipo ancestral A-C-C, cuya frecuencia estimada es del 52 % (0,5241). La importante frecuencia de este haplotipo podría favorecer la supervivencia de las neuronas estriatales durante episodios de toxicidad por mercurio, al mantener una actividad enzimática adecuada para la desintoxicación ^6^^,^^9^. De los 200 cromosomas en la población, se proyecta que 104 portarían esta combinación genética.

En contraste, los alelos mutantes rs1695-G 28, rs1138272-T ^29^ y rs6265-T ^30^, se han asociado con un mayor riesgo neurodegenerativo y con mayores concentraciones de mercurio. Resulta llamativo que el haplotipo N°6 (G-T-T), que reúne simultáneamente las tres variantes de riesgo, no se detectara en la muestra (frecuencia = 0; cuadro 2). Según las frecuencias alélicas individuales (cuadro 1), sería esperable la presencia, al menos, de un portador; su ausencia podría indicar un efecto selectivo negativo - probablemente deletéreo- que implique pérdida de función enzimática y, en consecuencia, mayor susceptibilidad a las toxinas. No obstante, no puede descartarse que el tamaño muestral haya sido insuficiente para observarlo.

En el cromosoma 13 se observaron dos haplotipos clave de *ATP7B.* El haplotipo GA (rs1061472-G / rs732774-A) aparece con una frecuencia de 0,5053 en nuestra muestra. Los alelos G y A, individualmente, se asociaron con menos mercurio en cabello, sangre u orina, tanto en profesionales dentales expuestos a vapores de amalgamas como en poblaciones ribereñas de la Amazonia con gran ingestión de pescado, probablemente porque facilita la exportación hepatobiliar de complejos Hg-GSH y reduce la bioacumulación ^6^^,^^31^. No obstante, el haplotipo TC (rs1061472-T / rs732774-C) presenta una frecuencia de 0,3936 y carece de ese efecto protector.

En el cromosoma 10, el haplotipo G-C-G (rs1885301-rs717620-rs2273697) del gen *ABCC2,* presenta una frecuencia estimada de 40 % (0,4043), lo que implica su presencia en unos 80 cromosomas de cada 200 en la población (≈ 100 individuos). Dado que el *ABCC2* codifica el transportador MRP2, esencial para la eliminación hepatorrenal de xenobióticos lipofílicos como el mercurio, esta prevalencia sugiere un posible impacto en la defensa frente al mercurio que traspasa la barrera hematoencefálica ^16^.

En los estudios sobre la luciferasa, se ha demostrado que el alelo rs1885301-G incrementa la actividad promotora y la expresión de *ABCC2,* lo cual favorece teóricamente la expulsión celular de mercurio ^32^. Por el contrario, en estudios poblacionales, se ha revelado que los portadores del alelo rs1885301-A exhiben concentraciones urinarias totales de mercurio ligeramente mayores y un desempeño neurológico más pobre, señal de una depuración sistémica menos eficiente ^33^. Así mismo, los portadores del alelo de riesgo rs717620-T, similar a los haplotipos de riesgo ATG o ATA en este estudio, muestran mayores concentraciones de mercurio urinario ^34^, ya que el alelo T provoca la pérdida de sitios de unión a los factores LEF-1 y SOX4, disminuyendo su expresión. En conjunto, estos hallazgos respaldan la idea de que la variabilidad en rs1885301 y rs717620 modula la toxicocinética del mercurio y, por ende, la susceptibilidad individual a sus efectos neurotóxicos, lo que justifica su inclusión en futuros paneles de riesgo genético.

La combinación alélica intercromosómica rs41303970-rs1050450-rs1202169-rs713041 (GGTC) de los genes *GCLM, GPX1, ABCB1* y *GPX4,* respectivamente, cuya frecuencia estimada es del 21 % (0,2129), agrupa variantes en cuatro locus clave y define un subgrupo poblacional potencialmente protegido frente a la toxicidad por mercurio. Esta protección se atribuye tanto al alelo rs1202169-T, que favorece el transporte renal y la eliminación del metal ^35^, como al alelo rs41303970-G, vinculado a una mayor actividad promotora de la subunidad modificadora del *GCLM* y, en consecuencia, a concentraciones aumentadas de glutatión ^36^.

En contraste, se identificaron cuatro combinaciones alélicas sin esta doble protección -AGCC, AACC, AACT y AGCT (cuadro 2)- que contienen al menos dos alelos de riesgo (rs41303970-A y rs1202169-C). La presencia del alelo rs41303970-A podría reducir la biosíntesis de glutatión y, por ende, la formación de complejos glutatión-mercurio, lo que dificultaría la excreción urinaria del metal, incrementando su carga renal y la vulnerabilidad antioxidante ^22^.

El análisis de redes reforzó la validez funcional de los polimorfismos identificados. Los genes *GSTP1* y el binomio *GCLC/GCLM* emergieron como genes centrales muy conectados, subrayando que variantes como rs1695, rs1138272 y rs1555903 podrían desestabilizar un eje antioxidante central en la conjugación glutatión-mercurio. Un segundo módulo integra metalotioneínas (MT1/2/3/4) con los transportadores *ATP7B* y *ABCC2/ ABCB1,* revelando un posible "cuello de botella" dual: la quelación intracelular y la exportación hepatobiliar del mercurio dependen de rutas que comparten SNP de riesgo frecuentes en la muestra de estudio.

Finalmente, la interconexión de *BDNF* con genes centrales antioxidantes vincula la homeostasis metálica con la plasticidad neuronal, lo que es coherente con la sensibilidad funcional descrita para rs6265. En conjunto, la red respalda una predisposición poligénica a la bioacumulación y toxicidad del mercurio en la población colombiana: la coincidencia de múltiples variantes en posiciones estratégicas amplifica el riesgo cuando la exposición ambiental es elevada.

El panorama genético delineado en este estudio revela que la población colombiana porta una gran cantidad de variantes asociadas con una mayor retención y toxicidad del mercurio. Entre estas variantes se destacan: a) los polimorfismos de las rutas antioxidantes GCLC rs17883901/rs1555903 y GSTP1 rs1695/rs1138272; b) los queladores intracelulares MT1A rs8052394 y rs11640851, y MT4 rs11643815; c) el BDNF rs6265 (Val66Met); y d) las bombas de transporte hepatorrenal ATP7B rs1061472 y rs732774, y ABCC2 rs1885301, rs717620 y rs2273697. En conjunto -y a menudo como haplotipos muy frecuentes (por ejemplo, GSTP1 ACC ≈ 52 %, ATP7B TC ≈ 39 %, ABCC2 GCG ≈ 40 %)-,, estas variantes sugieren una predisposición poblacional a la bioacumulación de mercurio y a efectos neurológicos y renales tempranos.

Aunque algunos haplotipos (por ejemplo, ATP7B GA) confieren cierta capacidad de depuración, la circulación simultánea de múltiples alelos de riesgo implica que una proporción sustancial de colombianos puede estar genéticamente expuesta a mayores cargas corporales frente a las fuentes comunes de mercurio: minería artesanal, amalgamas dentales e ingestión de pescado. Esta hipótesis exige validación mediante estudios de asociación en campo que midan de forma simultánea el genotipo, la especiación de mercurio, los marcadores clínicos (ß-2-microglobulina, tasa de filtración glomerular estimada, pruebas neurocognitivas) y los factores ambientales. El confirmar estas interacciones de los genes con el ambiente permitirá afinar los programas de vigilancia toxicológica, orientar intervenciones de salud pública y, en última instancia, diseñar estrategias de prevención personalizadas para las comunidades colombianas más expuestas.

En estudios futuros se buscará validar tanto la evidencia bibliográfica como los resultados del presente análisis de frecuencias génicas. Para ello, se llevarán a cabo muestreos de campo en poblaciones con exposición cotidiana al mercurio, contrastando la presencia de las variantes identificadas con la incidencia de enfermedades neurodegenerativas -Alzheimer y Parkinson- no solo en Antioquia, sino también en otros departamentos de Colombia.

Este estudio presenta varias limitaciones que deben tenerse en cuenta al interpretar los resultados. Primero, la cohorte analizada -94 individuos de Medellín pertenecientes al Proyecto 1000 Genomas- no garantiza una exposición comprobada al mercurio, lo que restringe la inferencia causal sobre los efectos reales de dicho metal. Además, el reducido tamaño de la muestra limita el poder estadístico para detectar asociaciones genéticas sutiles y aumenta la probabilidad de falsos negativos.

Tampoco se llevaron a cabo experimentos de validación funcional (expresión génica o estudios celulares) que confirmen la relevancia biológica de las variantes identificadas. La ausencia de información clínica directa, como diagnósticos de enfermedades neurodegenerativas o niveles sanguíneos de mercurio, impide relacionar los hallazgos genéticos con manifestaciones fenotípicas concretas. A ello se suma la falta de datos sobre otros factores ambientales o de estilo de vida que podrían influir en la expresión génica y en el riesgo neurodegenerativo, lo cual introduce posibles sesgos de confusión. Finalmente, el análisis estadístico se limitó a frecuencias y pruebas de x^2^, sin emplear modelos multivariados que ajusten simultáneamente por covariables relevantes, lo que reduce la solidez de las conclusiones.
